# Retracted: Oral Carcinogenesis and Oral Cancer Chemoprevention: A Review

**DOI:** 10.1155/2016/9267585

**Published:** 2016-06-28

**Authors:** Pathology Research International

The article titled “Oral Carcinogenesis and Oral Cancer Chemoprevention: A Review” [1] has been retracted as it was found to be essentially identical to the following published article: “Understanding Carcinogenesis for Fighting Oral Cancer” by Takuji Tanaka and Rikako Ishigamori in the Journal of Oncology.

## References

[1] Tanaka T., Tanaka M., Tanaka T. (2011). Oral carcinogenesis and oral cancer chemoprevention: a review. *Pathology Research International*.

